# Polysaccharides and Glycosides from *Aralia echinocaulis* Modulate Succinate Levels in the Gut to Target Intestinal Dendritic Cells via the Receptor GPR91 in the Treatment of Rheumatoid Arthritis

**DOI:** 10.3390/ph19040606

**Published:** 2026-04-09

**Authors:** Mengqiang Gao, Shanshan Ma, Yunzhi Li

**Affiliations:** School of Pharmacy, Anhui University of Chinese Medicine, Hefei 230012, China

**Keywords:** rheumatoid arthritis, *Aralia echinocaulis*, polysaccharides and glycosides, succinate, dendritic cells, GPR91

## Abstract

**Background**: *Aralia echinocaulis* has therapeutic effects on rheumatoid arthritis (RA), with total polysaccharide and glycoside (TPGs) as main active components. RA pathogenesis involves gut microbiota dysbiosis and immune–metabolic crosstalk, but the role of microbiota-derived succinate in RA remains unclear. **Objective**: This study explored the role of succinate-GPR91 signaling in intestinal dendritic cells (DCs) in the context of RA and the therapeutic mechanism of *A. echinocaulis* TPGs. **Methods**: Collagen-induced arthritis (CIA) mice were treated with TPGs or exogenous succinate. Paw edema, inflammation, gut succinate levels, the Th17/regulatory T (Treg) balance, and DC activation via succinate-GPR91 were detected, and GPR91-targeting siRNA and CD4+ T-cell coculture assays for verification. **Results**: TPGs alleviated symptoms in CIA mice and restored the Th17/Treg balance by reducing intestinal succinate levels. Succinate activated DCs via GPR91 to promote Th17 differentiation, while TPGs suppressed DC maturation and Th17-driven inflammation, supporting the involvement of a gut-centric immunometabolic axis in RA. **Conclusions**: TPGs ameliorate RA by targeting the succinate-GPR91-Th17 pathway, identifying succinate as a novel RA target and TPGs as a potential microbiota-modulating agent.

## 1. Introduction

Rheumatoid arthritis (RA) is a chronic autoimmune disease characterized by synovial inflammation and joint destruction and is driven by dysregulated immune responses involving autoreactive T cells, proinflammatory cytokines (e.g., tumor necrosis factor-α (TNF-α), interleukin (IL)-6, and IL-1β), and synovial fibroblast activation [1]. Despite advances in targeted therapies, the precise mechanisms linking environmental triggers and immune dysregulation in patients with RA remain incompletely understood. Emerging evidence highlights the role of metabolic intermediates, particularly succinate, in the modulation of inflammatory pathways. Succinate, which is traditionally viewed as a tricarboxylic acid (TCA) cycle metabolite, functions as an extracellular signaling molecule via its receptor GPR91 (SUCNR1) [2]. Activation of the succinate–GPR91 axis promotes proinflammatory cytokine (e.g., IL-6) production and exacerbates tissue inflammation in conditions such as obesity and colitis [3,4]. Intriguingly, GPR91 is highly expressed in dendritic cells (DCs), which are key antigen-presenting cells abundant on mucosal surfaces, including the gut [5]. These findings raise the possibility that gut-derived succinate may directly modulate systemic immune responses through DC activation. Notably, gut microbiota dysbiosis has been implicated in RA pathogenesis. Clinical and experimental studies have reported elevated levels of succinate-producing bacteria, particularly *Prevotella* species, in the intestines of RA patients and collagen-induced arthritis (CIA) models [6,7]. A *Prevotella*-enriched microbiota correlates with increased fecal and serum succinate levels, suggesting that microbes contribute to systemic succinate accumulation [8]. However, whether gut-derived succinate directly engages GPR91 on DCs to perpetuate joint inflammation remains unexplored. We hypothesize that dysbiosis of succinate-metabolizing microbes (e.g., *Prevotella* species) increases intestinal succinate production, which activates GPR91 on gut-resident DCs. This interaction triggers the secretion of IL-6 and other proinflammatory mediators, promoting systemic immune activation and synovial inflammation in patients with RA. By integrating metabolic and immunological axes, this study aims to elucidate a novel mechanism underlying RA pathogenesis and identify potential therapeutic targets.

**Figure 1:**
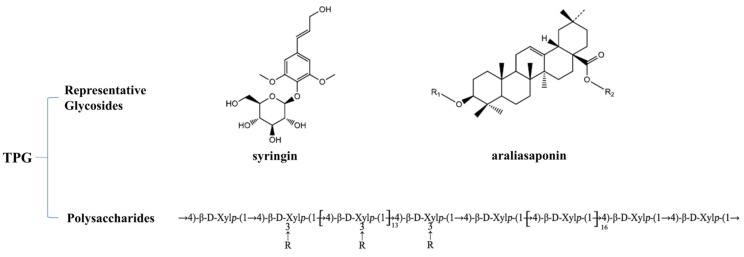
Representative chemical structures of the primary active constituents in TPGs.

*Aralia echinocaulis*, a medicinal and edible plant, has been traditionally used in parts of China as an alternative RA therapy because of its notable efficacy [9]. Our previous studies using CIA models demonstrated that total polysaccharides and glycosides (TPGs) from *A. echinocaulis* significantly ameliorated joint inflammation and cartilage damage, with gut microbiota modulation identified as a potential mechanism [10]. The representative chemical structures of the primary active constituents in TPGs, including glycosides such as syringin and araliasaponins, and the structural backbone of 4-O-methylglucuronoxylan are illustrated in Figure 1. However, whether TPGs exerts its anti-RA effects by targeting the succinate–GPR91 axis in intestinal DCs—a pathway implicated in microbial metabolite-mediated immune activation—remains unexplored.

Based on the background described above, this study employs animal and cellular models to investigate the role of the receptor GPR91 in intestinal DCs in RA pathogenesis and to elucidate the mechanisms of underlying the therapeutic effect of TPGs on RA.

While previous research has focused on the systemic anti-inflammatory properties of *Aralia echinocaulis*, the precise mechanism linking its microbiota-modulating effects to joint inflammation remains elusive. The scientific novelty of this study lies in uncovering the role of the intestinal succinate–GPR91 signaling axis as a pivotal mediator in the ‘gut–joint axis’. This study provides the first experimental evidence that TPGs ameliorates RA by suppressing succinate-induced DC maturation in the gut, thereby rectifying the systemic Th17/Treg imbalance. These findings contribute to the field by identifying gut-resident DCs as potential primary targets for herbal polysaccharides in the management of RA.

## 2. Results

### 2.1. Effects of TPGs and Succinate on Paw Edema

**Figure 2:**
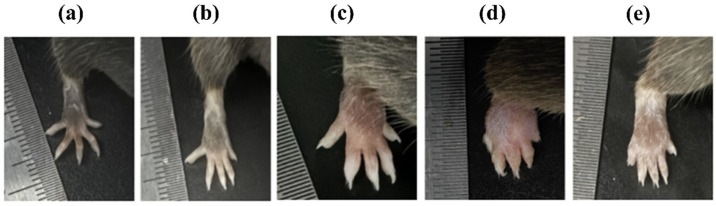
Photographs of the paws of mice from the different groups ((**a**) NG; (**b**) NG + succinate; (**c**) MG; (**d**) MG + succinate; (**e**) MG + TPGs). Notes: NG, normal control group; MG, model control group; TPGs, total polysaccharide and glycoside treatment group.

The changes in the body weight of the mice in each group during the experimental period are presented in Table S1. Following the induction of inflammation, the model mice presented with significant foot redness and swelling (Figure 2). The effects of TPGs on the severity of foot swelling and the foot circumference in the model mice are shown in Table 1 and Table 2. Treatment with TPGs for 24 days significantly attenuated the severity of swelling by approximately 28% compared with that in the model group. Compared with the NG, the MG exhibited a significant increase in paw thickness. In contrast, compared with the model mice, the TPGs-treated mice demonstrated a marked reduction in paw thickness after 24 days of treatment. Notably, foot swelling was significantly exacerbated, and the relative paw thickness was significantly greater in model animals treated with oral succinate for 24 days than in the untreated model mice, indicating the proinflammatory effect of succinate in this experimental model.

### 2.2. Effects of Succinate and TPGs on the Pathomorphology of the Joint Synovium in Model Mice

**Figure 3:**
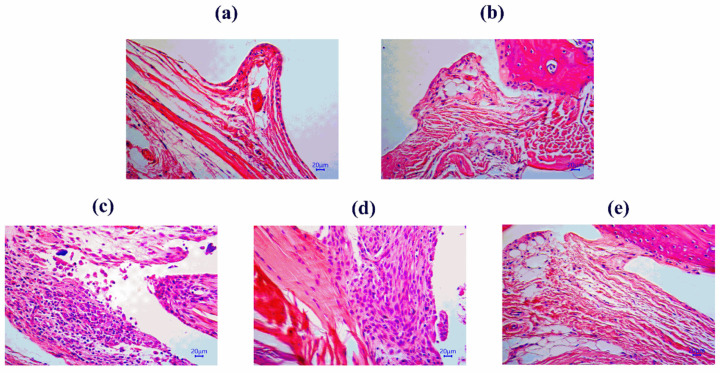
Effects of TPGs and succinate on the morphology of the ankle joint synovium in mice. ((**a**) NG; (**b**) NG + succinate; (**c**) MG; (**d**) MG + succinate; (**e**) MG + TPGs). Notes: NG, normal control group; MG, model control group; TPGs, total polysaccharide and glycoside treatment group.

To investigate the effects of TPGs and succinate on joint pathology, H&E staining of the ankle joint synovium was performed (Figure 3). The synovial architecture of the NG (Figure 3a) and NG + succinate group (Figure 3b) was normal and characterized by a thin synovial lining (1–2 cell layers) and the lack of inflammatory cell infiltration in the subsynovial stroma.

In contrast, the MG (Figure 3c) exhibited typical hallmarks of CIA, including significant synovial lining hyperplasia and marked leukocyte infiltration into the subsynovial tissue. This infiltration was characterized by a high density of small, dark-purple stained nuclei, indicating the recruitment of inflammatory cells such as neutrophils and mononuclear cells. Notably, in the MG + succinate group (Figure 3d), these pathological features were further exacerbated, with extensive and diffuse leukocyte recruitment that resulted in severe structural disorganization of the synovial membrane.

Conversely, the MG + TPG group (Figure 3e) demonstrated a substantial reduction in both synovial thickening and inflammatory cell density. Compared with that in the MG, the leukocyte infiltration in the TPG-treated mice was more localized and less dense, indicating that TPGs effectively restricted the recruitment of inflammatory cells to the joint, thereby protecting the structural integrity of the synovium.

### 2.3. Effects of Succinate and TPGs on Inflammatory Cytokine Levels in Serum and Intestinal Tissues Across Experimental Groups

**Figure 4:**
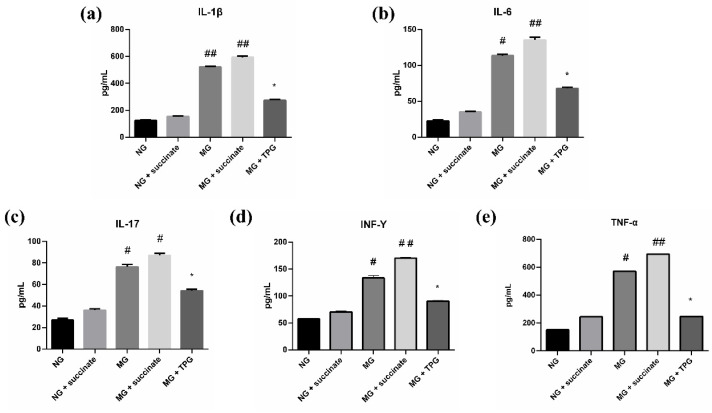
Effects of succinate and TPGs on the serum levels of inflammatory cytokines in the different groups of animals ((**a**) IL-1β; (**b**) IL-6; (**c**) IL-17; (**d**) INF-γ; (**e**) TNF-α). Compared with the NG, ^##^ *p* < 0.01, ^#^ *p* < 0.05; compared with the MG, * *p* < 0.05. *n* = 6. Notes: NG, normal control group; MG, model control group; TPGs, total polysaccharide and glycoside treatment group.

**Figure 5:**
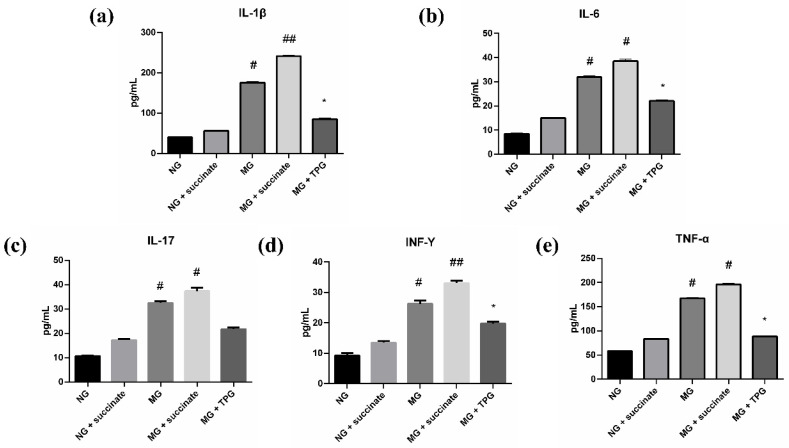
Effects of succinate and TPGs on the levels of inflammatory cytokines in the intestinal tissues of the different groups of animals ((**a**) IL-1β; (**b**) IL-6; (**c**) IL-17; (**d**) INF-γ; (**e**) TNF-α). Compared with the NG, ^##^ *p* < 0.01, ^#^ *p* < 0.05; compared with the MG, * *p* < 0.05. *n* = 6. Notes: NG, normal control group; MG, model control group; TPGs, total polysaccharide and glycoside treatment group.

As illustrated in Figure 4 and Figure 5, a comparative analysis of cytokine profiles revealed significant disparities among the experimental groups. Compared with the NG, the MG exhibited substantially increased serum and intestinal IL-1β, TNF-α, IL-6, IL-17, and IFN-γ levels. Specifically, the concentrations of these proinflammatory cytokines in the MG were significantly elevated, indicating the successful establishment of an inflammation model.

Subsequent treatment with succinate led to a further significant increase in the serum levels of IL-6, IFN-γ, and TNF-α, and the intestinal tissue levels of IL-1β and IFN-γ in the MG also notably increased (*p* < 0.01). These results suggest that succinate exacerbates the systemic inflammatory response, potentially by acting as a key mediator of disease progression. Conversely, TPG treatment exerted potent anti-inflammatory effects. Compared with the untreated MG, the TPG-treated group presented significantly lower expression levels of proinflammatory cytokines in both the blood and intestinal tissues. This attenuation of the inflammatory cascade indicates that TPGs have substantial therapeutic potential for the management of inflammatory conditions. These findings highlight the role of succinate in promoting the progression of the inflammatory response in animal models while underscoring the efficacy of TPGs as a promising therapeutic agent for inflammation-related disorders.

### 2.4. Effects of Succinate and TPGs on the Number of Neutrophils and MPO Activity in the Synovial Fluid of the Animals in Each Group

**Figure 6:**
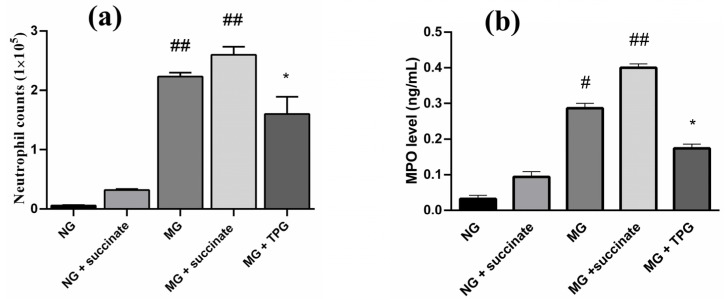
Effects of succinate and TPGs on neutrophil counts and MPO levels in the synovial fluid. (**a**) Neutrophil counts in the synovial fluid (×10^5^ cells). (**b**) MPO levels measured by ELISA. Compared with the NG, ^##^ *p* < 0.01, ^#^ *p* < 0.05; compared with the MG, * *p* < 0.05. *n* = 6. Notes: NG, normal control group; MG, model control group; TPGs, total polysaccharide and glycoside treatment group.

As shown in Figure 6, the number of neutrophils and the activity of MPO in the MG synovial fluid were significantly greater than those in the NG synovial fluid were (*p* < 0.05). Notably, TPG treatment significantly decreased these levels. In addition, MPO activity in serum and intestinal tissues was significantly greater in the succinate-treated group than in the MG without succinate treatment (*p* < 0.01), indicating that succinate induced inflammation.

### 2.5. Succinate Levels in Intestinal Tissues

**Figure 7:**
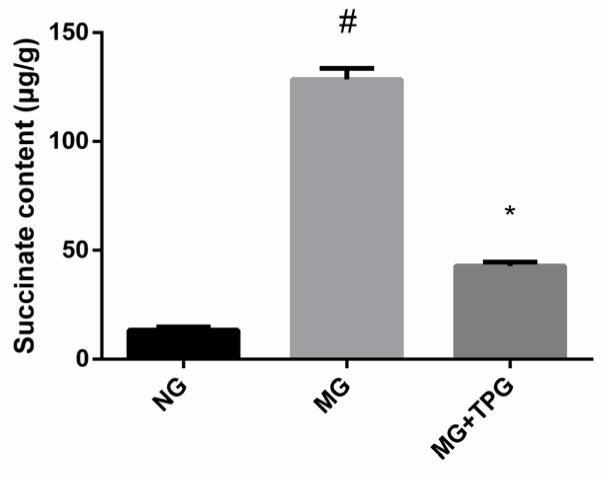
Concentrations of succinate in intestinal tissues in the control, model, and TPG intervention groups. Compared with the NG, ^#^ *p* < 0.05; compared with the MG, * *p* < 0.05. Notes: NG, normal control group; MG, model control group; TPGs, total polysaccharide and glycoside treatment group.

The above experiments demonstrated the proinflammatory effects of succinate and the anti-inflammatory effects of TPGs. Therefore, we next measured the levels of succinate in the intestinal tissues from the NG, MG, and TPG-treated group. As shown in Figure 7, compared with those in the NG, the succinate levels in the intestinal tissues in the MG were significantly greater. However, after TPG treatment, the levels of succinate in the intestinal tissues decreased significantly by 66.74%, from an average concentration of 128.4263 µg/g in the model group to 42.7187 µg/g in the TPG-treated group.

### 2.6. Percentages of Th17 and Treg Cells and the Th17/Treg Balance in Intestinal Tissues

**Figure 8:**
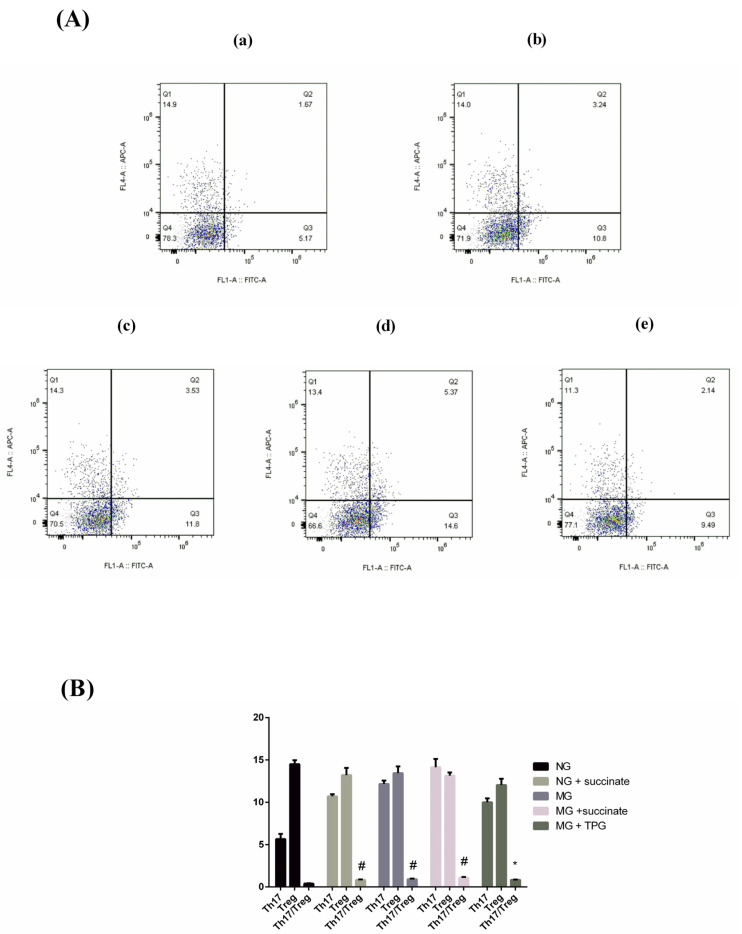
Effects of succinate on the Th17 and Treg cell populations in intestinal tissues across animal groups and following TPG treatment ((**A**) Representative flow cytometry plots: (**a**) NG; (**b**) NG + succinate; (**c**) MG; (**d**) MG + succinate; (**e**) MG + TPGs. LL: IL-17A^−^/Foxp3^−^, non-Th17/non-Treg cells; LR: IL-17A^+^/Foxp3^−^, Th17 cells; UL: IL-17A^−^/Foxp3^+^, Treg cells; UR: IL-17A^+^/Foxp3^+^, double-positive cells (usually a low proportion, nontargeted cell population; (**B**) Th17 and Treg cell frequencies. Compared with the NG, ^#^ *p* < 0.05; compared with the MG, * *p* < 0.05. *n* = 6). Notes: NG, normal control group; MG, model control group; TPGs, total polysaccharide and glycoside treatment group.

Previous studies have indicated that an imbalance between Treg cells and Th17 cells plays a crucial role in the pathogenesis of RA and triggers severe inflammatory responses. In the present study, flow cytometry was used to detect Th17 and Treg cells in the intestinal tissues of mice in the MG, and the ratio of the percentages of these two cell types was calculated. The results revealed that the Th17/Treg ratio in the intestinal tissues of the MG was significantly increased (Figure 8). We investigated the effect of succinate on the Th17/Treg ratio in both the NG and the MG. The findings revealed that the Th17/Treg ratio in intestinal tissues was significantly greater in the TPG-treated NG and MG than in the NG without succinate treatment. However, in the TPG-treated MG, the Th17/Treg ratio in intestinal tissues decreased significantly (Figure 8).

### 2.7. Immunophenotype of Intestinal DCs

**Figure 9:**
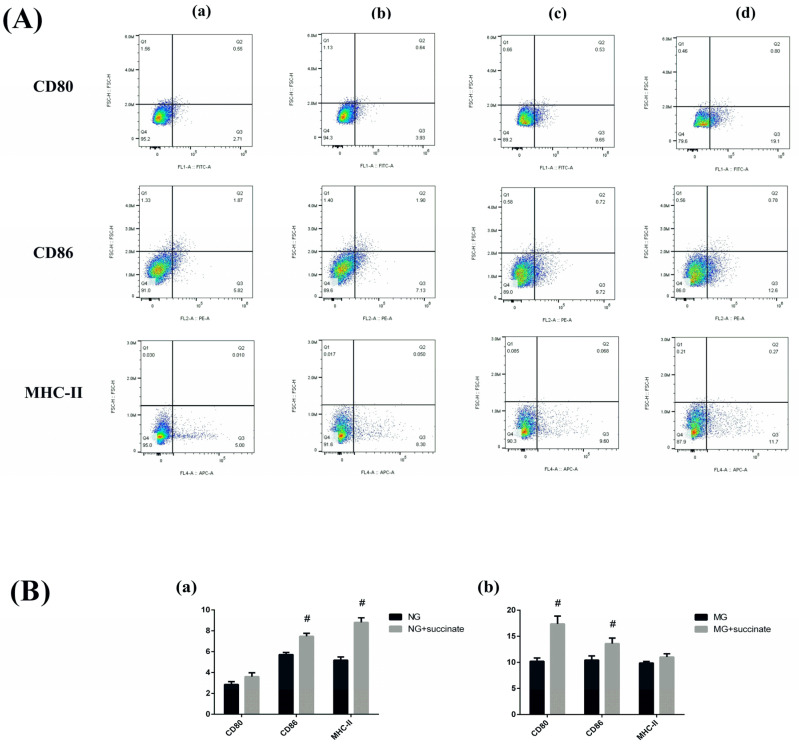
Effects of succinate on the immunophenotype of intestinal DCs ((**A**) Representative flow cytometry plots: (**a**) NG; (**b**) NG + succinate; (**c**) MG; (**d**) MG + succinate; (**B**) Effects of succinate intervention on CD80, CD86, and MHC-II expression in the NG (**a**) and MG (**b**)). Compared with the NG or MG, ^#^ *p* < 0.05. *n* = 6). Notes: NG, normal control group; MG, model control group; TPGs, total polysaccharide and glycoside treatment group.

MHC-II, CD80, and CD86 were expressed at very low levels in iDCs but were highly expressed in mDCs. To verify the ability of succinate to promote DC maturation, flow cytometry was used to determine the immunophenotype of intestinal DCs in each group. As shown in Figure 9, the expression levels of CD86 and MHC-II significantly increased in the group treated with succinate compared with those in the NG. In the MG, succinate treatment led to significant upregulation of CD80 and CD86 expression.

### 2.8. Succinate Exerts Its Effects Through GPR91

**Figure 10:**
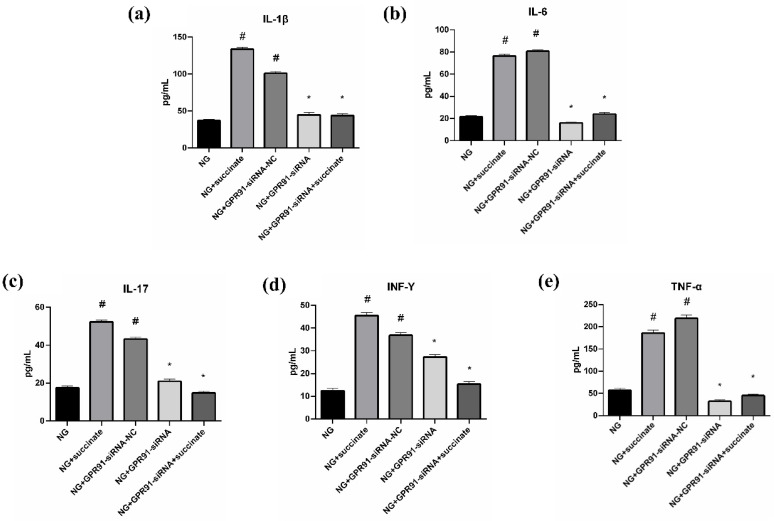
Succinate affects intestinal DCs through the receptor GPR91 ((**a**) IL-1β; (**b**) IL-6; (**c**) IL-17; (**d**) INF-γ; (**e**) TNF-α). Compared with the NG, ^#^ *p* < 0.05; compared with the NG + succinate, * *p* < 0.05, *n* = 6. Note: NG, normal control group.

To determine whether succinate affects DCs through GPR91, primary intestinal DCs from the NG were treated with a GPR91-specific small interfering RNA (siRNA). Afterward, double-antibody sandwich ELISAs were used to measure the protein expression levels of inflammatory cytokines, including IL-1β, TNF-α, IL-6, IL-17, and IFN-γ, in the cell culture supernatants before and after GPR91-targeting siRNA treatment. As depicted in Figure 10, succinate treatment significantly increased the expression levels of all of these inflammatory cytokines. Conversely, GPR91-targeting siRNA markedly reduced the expression levels of these cytokines. These results provide compelling evidence that succinate exerts its biological effects through the receptor GPR91 on DCs.

### 2.9. Coculture of Naïve T Cells with iDCs and mDCs In Vitro

**Figure 11:**
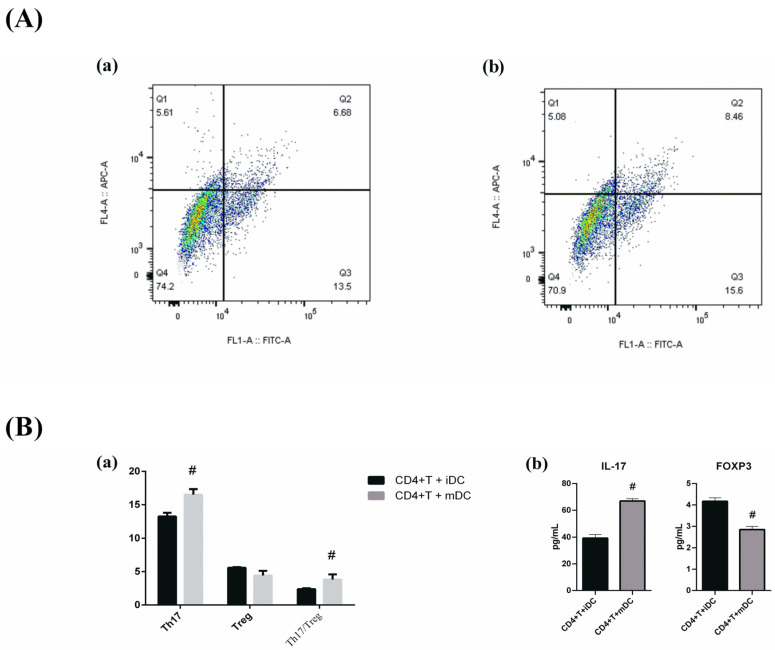
In vitro coculture of naïve T cells with two dendritic cell subsets (iDCs and mDCs) ((**A**) Representative flow cytometry plots of Th17 and Treg populations: (**a**) CD4+ T cells + iDCs; (**b**) CD4+ T cells + mDCs; (**B**) (**a**) percentages and ratios of Th17 and Treg cells; Specifically, the gray bars represent the CD4+ T cells + mDCs group, whereas the black bars represent the CD4+ T cells + iDCs group. (**b**) expression of Foxp3 and IL-17 in cell culture supernatants). Compared with CD4+ T cells + iDCs, ^#^ *p* < 0.05. *n* = 3.

To confirm the role of mDCs in RA pathogenesis, naïve T cells were cocultured in vitro with iDCs and mDCs. Flow cytometry was then used to determine the percentages of Th17 cells and Treg cells. ELISAs were used to measure the expression levels of Foxp3 and IL-17 in the cell culture supernatants. The results are presented in Figure 11. These findings demonstrated that coculture of mDCs with naïve T cells facilitated the generation of Th17 cells, increased the expression of IL-17, and decreased the expression of Foxp3.

## 3. Discussion

RA is a chronic autoimmune disorder with a substantially increasing global burden, with a global prevalence of 0.3–2%. Epidemiological data from the World Health Organization [11] indicate that approximately 18 million individuals were affected by RA in 2019. In China, the estimated prevalence is 0.42%, which corresponds to nearly 5 million individuals. RA is characterized by persistent synovial inflammation, progressive joint damage, and fluctuating disease activity. It is typically a lifelong disease that poses significant clinical and socioeconomic challenges.

Although therapeutic advancements—including biologic disease-modifying antirheumatic drugs (bDMARDs) and targeted synthetic disease-modifying antirheumatic drugs (tsDMARDs)—have substantially improved disease management, critical limitations persist. These include heterogeneous treatment responses, secondary drug resistance, adverse effects associated with long-term immunosuppression, and the considerable economic burden of prolonged therapy. Consequently, RA remains an unresolved clinical challenge in the field of rheumatology, necessitating further research into novel therapeutic strategies and precision medicine approaches.

The pathogenesis of RA is complex and multifactorial, and involves genetic predispositions, environmental triggers, and dysregulated immune responses [12]. Emerging evidence highlights the critical interplay between metabolic reprogramming and inflammatory processes in autoimmune diseases, and metabolites such as succinate act as key immunomodulators [13]. Notably, elevated succinate levels in fecal and synovial fluid samples from RA patients suggest its role in disease progression [14,15]. As a hub metabolite that links mitochondrial respiration, the TCA cycle, and extracellular signaling, succinate activates GPR91 (SUCNR1) to regulate innate immunity, tissue remodeling, and cytokine storms [4,5,16,17,18,19,20].

Intriguingly, mucosal tissue—particularly gut-associated lymphoid tissue—has emerged as a potential site of RA initiation. Autoantibodies such as IgA-ACPAs emerge years before the onset of clinical joint symptoms, indicating that intestinal dysbiosis is involved in early immune dysregulation [21]. DCs densely populate the intestinal lamina propria and act as sentinels linking microbial metabolites to adaptive immunity [22]. Studies have demonstrated that succinate-GPR91 signaling enhances DC antigen presentation, promotes TLR-driven proinflammatory cytokine (e.g., TNF-α and IL-1β) production, and skews T-cell polarization toward Th17 dominance [5,23]. These effects are abolished in GPR91-deficient DCs, underscoring the pivotal role of this receptor in RA-related inflammation [5,23].

Accumulating evidence has linked alterations in the gut microbiota to RA pathogenesis. For example, the overgrowth of *Prevotella copri* in treatment-naïve RA patients is correlated with increased disease severity and a decline after therapy [6]. Mechanistically, *P. copri*-enriched fecal transplantation exacerbates arthritis in SKG mice via the expansion of intestinal Th17 cells [24]. *P. copri* uniquely stimulates DCs to produce the cytokines IL-6, IL-17, and IL-23, which are critical for Th17 differentiation [24]. Critically, *P. copri* is a potent succinate producer; its colonization in germ-free mice increases intestinal succinate levels, establishing a direct microbiota–metabolite–immune axis in the context of RA [25].

In recent years, increasing attention has been given to the role of herbal polysaccharides in modulating the composition and function of the gut microbiota. These bioactive macromolecules exhibit diverse pharmacological properties while maintaining a favorable safety profile, positioning them as promising therapeutic agents for the prevention and treatment of chronic diseases. Accumulating evidence has demonstrated that herbal polysaccharides can selectively promote the proliferation of beneficial microbial species (e.g., *Bifidobacterium* and *Lactobacillus*) while suppressing pathogenic bacteria, thereby restoring gut microbial homeostasis. This microbiota-regulating capacity underlies their therapeutic efficacy in various chronic conditions, including inflammatory bowel disease, obesity, type 2 diabetes mellitus, hepatic disorders, and cancer.

*A. echinocaulis* (Araliaceae) is a perennial medicinal plant widely distributed across southeastern China, including Zhejiang, Anhui, Jiangxi, Hunan, Guangdong, and Guangxi Provinces. In the Enshi region, this species has been extensively employed in traditional medicine for the management of musculoskeletal disorders, particularly Bi syndrome (rheumatoid conditions), fracture healing, and traumatic injuries, with well-documented therapeutic efficacy. Our prior work revealed the efficacy of *A. echinocaulis*-derived TPGs in ameliorating joint inflammation via gut microbiota modulation, but its interaction with the succinate–GPR91 axis remains unexplored.

Given the background presented above, this study employed both animal and cellular models to investigate the role of the intestinal DC receptor GPR91 in RA pathogenesis and to elucidate the mechanisms underlying the antiarthritic effect of TPGs.

First, we successfully established a CIA mouse model and administered succinate and TPGs. Consistent with previous reports, TPGs exerted significant therapeutic effects in the RA model, markedly ameliorating paw swelling, decreasing plantar thickness, and normalizing synovial histomorphology. In contrast, disease progression was exacerbated in the succinate-treated group compared with the MG. Similarly, the serum and intestinal levels of proinflammatory cytokines (IL-1β, TNF-α, IL-6, IL-17, and IFN-γ) were significantly reduced in the TPG-treated group, whereas succinate stimulation led to notable increases in the levels of some inflammatory mediators. The above experimental data indicate that TPGs have significant therapeutic effects on a mouse model of RA and can effectively alleviate the symptoms of this disease. In contrast, succinate significantly exacerbates the inflammatory response in this animal model and promotes disease progression.

Building upon the cytokine profiling results, we further investigated the effects of TPGs and succinate on the Th17/Treg balance in intestinal tissues. The experimental data demonstrated that succinate significantly increased the Th17/Treg ratio in both the NG and the MG, whereas TPG treatment effectively restored immune homeostasis. Emerging evidence highlights the pivotal role of Th17/Treg cell imbalance in the pathogenesis of RA. As key immunoregulatory subsets, proinflammatory Th17 cells and immunosuppressive Treg cells perform reciprocal functions in maintaining immune homeostasis. Notably, RA patients consistently exhibit an elevated Th17/Treg ratio, characterized by an increased percentage of Th17 cells and a decreased percentage of Treg cells, which may perpetuate chronic inflammation and drive disease progression [26,27,28]. Restoration of the Th17/Treg balance has emerged as a promising therapeutic strategy for treating RA patients. Mechanistic studies have revealed that PTEN ameliorates autoimmune arthritis by normalizing the Th17/Treg balance through the inhibition of STAT3 signaling [29]. Similarly, cyanidin has shown therapeutic potential in RA models because it rebalances Th17/Treg populations via ROCK2 pathway modulation while concurrently suppressing T follicular helper cell differentiation [30]. Recent advances have identified microRNAs as critical regulators of the Th17/Treg balance. In particular, decreased miR-21 expression is correlated with the dysregulation of the Th17/Treg balance in RA patients. Targeted modulation of miR-21 levels may represent a novel therapeutic approach to restore immune balance [31]. These findings collectively underscore the central importance of the Th17/Treg balance in both RA pathogenesis and the development of RA treatments. Further investigations into the molecular mechanisms underlying this equilibrium may yield innovative immunomodulatory therapies for the management of RA. Our study demonstrated that TPG can effectively regulate the Th17/Treg balance, whereas succinate exacerbates this imbalance, indicating the proinflammatory effect of succinate.

Neutrophil infiltration and MPO activity in synovial fluid play pivotal roles in RA pathogenesis. Recent evidence indicates that MPO levels are significantly elevated in the synovial fluid of RA patients and are strongly correlated with inflammatory processes [32]. Through its catalytic properties, MPO promotes neutrophil recruitment and activation, thereby exacerbating joint inflammation [33]. In addition to its proinflammatory functions in RA, MPO has been identified as a mediator of local tissue damage in experimental arthritis models. Notably, MPO-deficient mice exhibit decreased severity of experimental arthritis, underscoring the pathogenic contribution of MPO to disease progression [34]. These findings highlight the pathophysiological importance of MPO in RA and suggest its potential as a therapeutic target. On the basis of this evidence, in addition to performing cytokine profiling, we assessed neutrophil infiltration and MPO activity in synovial fluid, and both were elevated in the model group but decreased by TPG treatment. Conversely, succinate further increased MPO activity. These findings strongly suggest a pathogenic link between succinate and the development of RA.

Given that GPR91 is highly expressed in mDCs, we isolated intestinal DCs from the experimental groups and analyzed their immunophenotypes (by measuring MHC-II, CD80, and CD86 expression) via flow cytometry. The levels of these surface markers, which are typically low in iDCs but elevated in mDCs, significantly increased upon succinate stimulation in both normal and CIA model mice, indicating that succinate promotes DC maturation. Furthermore, we examined intestinal Th17 and Treg populations and found that succinate skewed the Th17/Treg balance toward a proinflammatory state, reinforcing its role in driving intestinal immune dysregulation.

To assess whether succinate exerts its effects via GPR91, we performed siRNA-mediated GPR91 knockdown experiments. Flow cytometric analysis confirmed that succinate-induced DC maturation (as evidenced by increased MHC-II, CD80, and CD86 expression) was abolished upon GPR91 silencing. Additionally, ELISAs revealed that GPR91 knockdown suppressed the succinate-induced secretion of IL-1β, TNF-α, IL-6, IL-17, and IFN-γ, suggesting that succinate activates DCs and promotes inflammation through GPR91 signaling.

Emerging evidence highlights the pivotal role of mDCs in driving the differentiation of CD4+ T cells into Th17 cells. mDCs orchestrate Th17 polarization through both cytokine-dependent and cell contact-mediated mechanisms. Specifically, mDCs secrete key polarizing cytokines, including IL-1β, IL-6, and IL-23, which are essential for initiating and sustaining Th17 differentiation [35]. In addition to soluble factors, direct interactions between mDCs and CD4+ T cells further amplify Th17 cell generation. Notably, under pathological conditions such as Crohn’s disease, mDCs can activate NKG2D receptor signaling in CD4+ T cells, triggering excessive production of IL-17 and IL-22 and thereby exacerbating Th17-mediated inflammation [36]. These findings suggest that mDCs employ context-dependent pathways to modulate Th17 responses. Moreover, cross-talk between mDCs and other immune cells fine-tunes Th17 development. For example, interactions between mDCs and monocyte-derived dendritic cells can modulate IL-17 and IL-10 production via CTLA-4 signaling, demonstrating the complexity of Th17 regulation in inflammatory microenvironments [37]. In summary, mDCs promote Th17 differentiation through multiple mechanisms involving cytokine secretion, direct cellular contact, and intercellular crosstalk. Deciphering these pathways provides critical insights into Th17 biology and its contribution to inflammatory diseases, offering potential therapeutic targets for immune-mediated pathologies.

Foxp3, the master transcriptional regulator of Treg cells, plays a pivotal role in maintaining immune tolerance and suppressing autoimmune responses. In RA patients, both the expression and the functionality of Foxp3 are frequently impaired. Intriguingly, studies have revealed significant upregulation of both full-length and spliced Foxp3 mRNA isoforms in peripheral blood CD4+ T cells from RA patients [38]. This observation suggests the potential existence of distinct T-cell subpopulations that may be selectively recruited to inflamed joints during disease progression. Moreover, emerging evidence has demonstrated the crucial involvement of Foxp3 in the pathogenesis of rheumatoid arthritis. The regulation of Foxp3 expression involves complex mechanisms, particularly epigenetic modifications. Notably, compared with healthy controls, patients with RA exhibit significantly reduced Foxp3 expression and decreased demethylation at the Treg-specific demethylated region (TSDR) [39], highlighting the crucial role of epigenetic dysregulation in RA pathogenesis. Furthermore, conventional disease-modifying antirheumatic drugs (DMARDs) have been shown to ameliorate disease activity and inflammation in patients with RA by upregulating the expression of Foxp3 along with the expression of the Sema-3A and Nrp-1 genes [40]. The therapeutic modulation of Foxp3 expression represents a promising research avenue for treating RA patients. In particular, methotrexate (MTX) therapy can restore Treg function in RA patients through the demethylation of the Foxp3 upstream enhancer region, consequently reducing disease activity [41]. Collectively, these findings underscore the central importance of Foxp3 in both RA pathogenesis and treatment, suggesting that further investigation into Foxp3-related mechanisms may yield novel therapeutic targets and strategies for the management of RA.

On the basis of this background information, we cocultured intestinal mDCs with naïve CD4+ T cells and observed increased Th17 cell generation, as characterized by increased IL-17 production and reduced Foxp3 expression. These findings provide mechanistic insight into how intestinal DC activation may contribute to joint inflammation in patients with RA.

In recent years, interest in the involvement of the succinate–GPR91 signaling axis in RA has increased. Mounting evidence indicates that macrophage activation under inflammatory conditions leads to metabolic reprogramming and subsequent succinate accumulation in SUCNR1/GPR91-expressing macrophages. These activated macrophages not only release succinate into the extracellular milieu but also upregulate GPR91 expression. As an autocrine/paracrine sensor, GPR91 detects extracellular succinate and amplifies IL-1β production. Notably, GPR91 deficiency significantly attenuated macrophage activation and IL-1β secretion in antigen-induced arthritis models [42]. Clinical observations have revealed elevated succinate levels in the synovial fluid of RA patients, which potently induces GPR91-dependent IL-1β release from macrophages. These findings reveal a GPR91/succinate-mediated positive feedback loop that perpetuates macrophage activation and sustains inflammatory responses. These mechanistic findings suggest that GPR91 antagonists are promising therapeutic candidates for the management of RA [42].

In summary, this study has several notable strengths. C57BL/6 mice were selected as the experimental model because of their stable genetic background, high susceptibility to collagen-induced arthritis (CIA) model establishment, and widespread application in immunometabolic and arthritis-related mechanistic research; this selection ensured the stability and reproducibility of the experimental results and facilitated direct comparison of our findings with those of previously published related studies. We provide the first evidence linking intestinal homeostasis to articular inflammation via the succinate-GPR91 signaling axis, offering a novel “gut–joint axis” perspective for RA treatment. Furthermore, the therapeutic efficacy of TPGs was validated through both in vivo animal models and in vitro DC–T-cell coculture systems, ensuring the robustness of our findings. However, several limitations must be acknowledged. While our in vitro siRNA knockdown experiments and in vivo observations revealed a strong association between the succinate-GPR91 axis and the effects of TPGs, these results do not establish a definitive causal relationship. The systemic nature of immune regulation suggests that other parallel signaling pathways may also be involved. Therefore, future research employing GPR91-knockout (*Sucnr*1^−/−^) mice or specific GPR91 antagonists is necessary to rigorously confirm the essentiality of this receptor in TPG-mediated protection. Additionally, although we used male mice to ensure model stability and reproducibility, the potential influence of sex-based hormonal variations remains to be explored. Finally, future work utilizing metagenomic sequencing will be essential to precisely identify the specific succinate-producing bacterial taxa modulated by TPGs. Addressing these gaps will aid in the development of more targeted, microbiota-directed strategies for RA management.

## 4. Materials and Methods

### 4.1. Preparation of TPGs

TPGs were prepared as previously described [10]. Briefly, *A. echinocaulis* roots (voucher No. lyz09; Wenzhou, China, June 2021) were processed to isolate glycosides and polysaccharides. Glycosides were extracted from root bark with 85% ethanol and sequentially partitioned with petroleum ether, ethyl acetate, and *n*-butanol. The *n*-butanol fraction was purified with D101 macroporous resin (90% ethanol elution, 18:1 height/diameter ratio) and Sephadex LH-20 column chromatography (50% methanol), achieving a purity of 96.9%. Polysaccharides were isolated from the ethanol-extracted residue through aqueous extraction (10:1 water-to-material ratio), ethanol precipitation (4:1 ethanol-to-supernatant ratio), decolorization (with 1% activated carbon), deproteinization (Sevag method), and lyophilization, yielding a final purity of 96.0%. The purified glycosides and polysaccharides were combined to yield TPGs for subsequent use. (The detailed preparation workflow is illustrated in Supplementary Figure S1). Previous studies have demonstrated that TPGs include characteristic glycosides including syringin, araliasaponins IV, VI, VII, and XVI, as well as a polysaccharide identified as 4-*O*-methylglucuronoxylan (molecular weight: 16 kDa) composed of xylan and 4-*O*-methylglucuronic acid [43,44,45].

### 4.2. Establishment of the CIA Model and Experimental Groups

Forty-two male C57BL/6 mice (specific pathogen-free grade, 20–25 g) were acclimatized for one week under controlled environmental conditions (temperature: 22–25 °C; relative humidity: 40–70%) prior to experimentation. CIA was established in 24 mice by subcutaneous injecting 0.1 mL of an emulsion containing equal volumes (50 μL each) of chicken type II collagen (2 mg/mL; Chondrex, Inc., Woodinville, WA, USA) and complete Freund’s adjuvant (containing 2 mg/mL *M. tuberculosis*; Chondrex, Inc., Woodinville, WA, USA), for a final dose of 100 μg of collagen per mouse. A booster immunization was administered on day 21 after the primary immunization. Successful model establishment was confirmed on day 16 after the booster immunization, and 18 successful modeled animals were randomly assigned to three experimental groups (*n* = 6/group) using a computer-generated random number table to ensure unbiased allocation: the model control group (MG), the MG + succinate group, and the MG + TPG treatment group. Twelve control mice were not immunized and were assigned to one of two control groups (*n* = 6/group): the normal control group (NG) and the NG + succinate group. The sample size (*n* = 6 per group) was determined on the basis of a power analysis using G*Power software (version 3.1.9.7), as described in our previous study [10]. With an anticipated effect size (Cohen’s *d*) of 1.8 for the primary outcome (plantar swelling), a sample size of *n* = 6 provided a statistical power of 0.8 with a significance level of 0.05. This also aligns with the 3R principles of animal ethics, which aim to minimize animal usage while ensuring robust statistical data.

All the experimental groups were administered the corresponding treatments via daily oral gavage different dosages and for different durations: the MG and NG were given 0.1 m of normal saline each day as the vehicle control for 24 consecutive days; the MG + succinate and NG + succinate groups were treated with succinate at a dosage of 800 mg/kg/day for 14 consecutive days; and the MG + TPG group was administered TPGs at a dosage of 800 mg/kg/day for 24 consecutive days. The dose, route, and frequency of TPG administration were based on our previous dose–response study [10], where 800 mg/kg/day was identified as the optimal dosage for achieving significant therapeutic efficacy, and only this single optimal dose was adopted in the present study to focus on the underlying immunometabolic mechanism exploration in line with the 3Rs principle for animal welfare. The dosage, administration route and duration of succinate were set according to a previously described protocol [46]. the following parameters were evaluated during the experiment:

(1) Body weight: Body weight was measured at baseline (day 0) and on treatment days 6, 12, 18, and 24.
(2) Plantar thickness: Right hind paw thickness was measured using digital calipers before modeling and at 6-day intervals after treatment. The swelling percentage was calculated as follows:
  Plantar swelling (%) = (postimmunization thickness − baseline thickness)/baseline thickness × 100.
(3) Paw edema: The volume of the ankle joint (including distal structures) was quantified via the water displacement method before modeling and at specified intervals during treatment. The percentage of edema was calculated as follows:
  Paw edema (%) = (postimmunization volume − baseline volume)/baseline volume × 100.

After 24 days of treatment, mice were anesthetized via intraperitoneal injection of 1% sodium pentobarbital (0.1 mL/20 g). Blood was collected from the abdominal aorta for cytokine analysis. The ankle joints, synovium, and intestinal tissues (jejunum, ileum, and colon) were subsequently harvested. For the ankle joints, 4% paraformaldehyde (PFA) was injected into the joint cavities for in situ fixation prior to immersion in the same fixative. All harvested tissues were fixed in 4% PFA for 8 h, followed by decalcification (for joints), sequential dehydration, and paraffin embedding. Sections (6 μm) were prepared and stained with hematoxylin and eosin (H&E) for histopathological evaluation via light microscopy. The animal carcasses were disposed of by the Laboratory Animal Center of Anhui University of Chinese Medicine in accordance with institutional guidelines. Biological specimens were then subjected to the following specific analyses:

(1) Histopathological evaluation of the ankle joint synovium: H&E-stained sections of the right hind ankle joints were examined to assess synovial inflammation and tissue damage.

(2) Quantification of cytokine levels in serum and intestinal tissues: Blood collected from the abdominal aorta was centrifuged (3500 rpm, 10 min) to obtain serum. For intestinal tissues, segments of the jejunum, ileum, and colon (approximately 50 mg each) were homogenized in cold PBS containing a protease inhibitor cocktail, followed by centrifugation (12,000 rpm, 15 min, 4 °C), and the supernatant were collected. The total protein concentration in the tissue supernatants was determined using a BCA protein assay kit (Thermo Fisher Scientific, Waltham, MA, USA). The levels of IL-1β, TNF-α, IL-6, IL-17, and IFN-γ in both serum and tissue supernatants were quantified using commercial ELISA kits (ELK Biotechnology Co., Ltd., Wuhan, China) according to the manufacturer’s instructions. The tissue cytokine levels were normalized to the total protein content (pg/mg protein).

(3) Synovial fluid neutrophil numbers and measurement of myeloperoxidase (MPO) activity: To count neutrophils, the joint cavities were lavaged with 1 mL of PBS, and the collected synovial fluid was smeared onto glass slides. The slides were stained with Wright–Giemsa solution (2–3 drops) for 1–2 min, after which an equal volume of 0.01 M phosphate buffer (pH 6.4–6.8) was added for 3–5 min. After the slides were washed and dried, the neutrophils were counted under a light microscope. MPO levels in the synovial fluid were measured using a commercial ELISA kit (WEIAO Biotechnology Co., Ltd., Shanghai, China) according to the manufacturer’s instructions.

(4) Quantification of succinate levels: HPLC analysis of succinate concentrations in intestinal tissue was performed. To analysis the tissue samples, approximately 100 mg of the sample was homogenized in 200 μL of 5% aqueous perchloric acid (4 °C, 300 s) in microcentrifuge tubes. The homogenates were subjected to extraction and centrifugation procedures identical to those described for the serum samples.

Analyses were performed on an Agilent 1200 series HPLC system equipped with a C18 reverse-phase column (Agilent Technologies, Santa Clara, CA, USA) (250 mm × 4.6 mm, 5 μm particle size). The mobile phase consisted of 0.1 mol/L sodium sulfate buffer (pH 2.65) delivered at a flow of 1.0 mL/min. Detection was achieved using a variable wavelength detector set at 214 nm, with the column temperature maintained at 30 °C. The samples were introduced via a 30 μL injection loop. Method validation confirmed that the filtration step resulted in a >98% protein precipitation efficiency and 99.2–101.5% analyte recovery, and the reproducibility of the retention time was <0.8% RSD across six consecutive runs.

### 4.3. Isolation of Intestinal DCs and Naïve CD4+ T Cells

#### 4.3.1. Isolation of Intestinal DCs

Jejunum, ileum, and colon tissues were collected, thoroughly rinsed with PBS, and cut into approximately 0.5 cm pieces. DCs in the intestinal lamina propria were then isolated using magnetic-activated cell sorting (MACS).

#### 4.3.2. Isolation of Naïve CD4+ T Cells from the Intestinal Tissue of Mice in the NG

Intestinal tissues were opened, rinsed with PBS, and stripped of the epithelial layers and adipose tissue. The tissues were then minced and digested in a solution containing 4% fetal bovine serum (FBS), 1 mg/mL collagenase, 0.5 mg/mL dispase, and 40 μg/mL DNase at 37 °C for 50 min in a shaking water bath. The resulting cell suspension was enriched via Percoll density gradient centrifugation (40%/80%). CD4+ T cells were further purified using anti-CD4 magnetic microbeads and resuspended in complete RPMI 1640 medium supplemented with 10% FBS.

The following parameters were analyzed:

(1) DC immunophenotype: The surface expression of major histocompatibility complex class II (MHC-II), cluster of differentiation 80 (CD80), and cluster of differentiation 86 (CD86) was assessed via flow cytometry.
(2) Th17 and Treg cell proportions: The percentages of Th17 and Treg cells in intestinal tissues were determined via flow cytometry.

#### 4.3.3. Grouping and Treatment of Intestinal DCs from the NG

Primary intestinal DCs were isolated from mice in the NG and cultured for 72 h before they received one of the treatments indicated in Table 3. The single concentrations of succinate and GPR91-targeting siRNA used in the table were selected on the basis of our previous preliminary dose–response experiments and were identified as the optimal concentrations to induce significant regulatory effects on intestinal DC biological functions.

The following parameters were measured:

(1) DC immunophenotype: MHC-II, CD80, and CD86 expression levels were measured via flow cytometry.
(2) Inflammatory cytokine levels: The concentrations of IL-1β, TNF-α, IL-6, IL-17, and IFN-γ in the cell culture supernatants were quantified using sandwich ELISA.

### 4.4. Coculture of Naïve CD4+ T Cells with DCs

Mature dendritic cells (mDCs) were generated by treating immature DCs (iDCs) isolated from the intestinal lamina propria with recombinant murine TNF-α (20 ng/mL) for 24 h. CD11c+ cells were purified using immunomagnetic beads through positive selection. DC maturation status was confirmed by flow cytometry analysis of surface markers (CD80/CD86/MHC II).

Naïve CD4+ T cells were cocultured with DCs (iDCs or mDCs) in triplicate wells for 120 h. Afterward, the following analyses were performed:

(1) Flow cytometric analysis: The percentages of Th17 (CD4+IL-17A+) and regulatory T (Treg, CD4+CD25+Foxp3+) cells were quantified using fluorescently labeled antibodies against specific surface and intracellular markers.
(2) Cytokine profiling: Cell culture supernatants were collected and subjected to ELISA to measure forkhead box P3 (Foxp3) and interleukin-17 (IL-17A) expression levels according to the manufacturer’s instructions.

### 4.5. Statistical Analysis

Statistical analyses were performed with GraphPad Prism software (version 6.0). Continuous data are expressed as the means ± standard deviations (SDs). The significance of intergroup differences was determined by one-way ANOVA with Tukey’s post hoc test for multiple comparisons. The threshold for statistical significance was defined as *p* < 0.05. Significant differences between groups are indicated with distinct markers, as defined in the corresponding figure legends. In general, ^#^/^##^ denote *p* < 0.05/*p* < 0.01 compared with the indicated control group (e.g., NG or CD4+ T + iDC), while */** denote *p* < 0.05/*p* < 0.01 compared with the MG or other indicated comparison groups.

## 5. Conclusions

This study provides the first experimental evidence that the succinate-GPR91 axis drives RA pathogenesis through gut-centric immunometabolic mechanisms. Mechanistically, intestinal succinate accumulation promotes DC maturation via GPR91 activation, thereby skewing T-cell polarization toward Th17 dominance and increasing the levels of systemic proinflammatory cytokines (IL-1β, TNF-α, IL-6, IL-17 and IFN-γ). Crucially, the anti-RA effects of TPGs are partially mediated by the decreased production of succinate, highlighting this metabolite as a novel therapeutic target. Our findings reveal a pathogenic cascade linking gut microbiota-derived succinate to extra-articular immune dysregulation, providing insights into the development of microbiota-directed RA therapies.

## Figures and Tables

**Table 1:** Effects of TPGs and succinate on the thickness of the hind paw in mice with collagen-induced arthritis (mean ± SD, %, *n* = 6).

Group	6 d	12 d	18 d	24 d
NG	13.43 ± 1.90	13.54 ± 3.00	14.14 ± 2.95	14.94 ± 3.02
NG + succinate	14.00 ± 0.99	14.63 ± 2.01	14.73 ± 2.15	15.23 ± 2.44
MG	94.07 ± 17.73 ^##^	101.74 ± 7.48 ^##^	102.90 ± 5.25 ^##^	94.28 ± 9.44 ^##^
MG + succinate	111.40 ± 3.91 *	117.45 ± 5.52 **	112.26 ± 4.74 **	106.69 ± 5.30 **
MG + TPGs	85.82 ± 9.79	81.86 ± 8.47 **	78.43 ± 6.67 **	70.13 ± 4.27 **

Notes: Compared with the NG, ^##^ *p* < 0.01; compared with the MG, ** *p* < 0.01, * *p* < 0.05. NG, normal control group; MG, model control group; TPGs, total polysaccharide and glycoside treatment group.

**Table 2:** Effects of TPGs and succinate on the degree of paw swelling in mice with collagen-induced arthritis (mean ± SD, %, *n* = 6).

Group	6 d	12 d	18 d	24 d
NG	16.85 ± 4.01	15.58 ± 2.90	16.59 ± 2.38	17.86 ± 3.29
NG + succinate	16.37 ± 3.77	13.98 ± 0.75	15.10 ± 2.29	17.34 ± 2.69
MG	86.74 ± 19.71 ^##^	84.39 ± 15.1 ^##^	97.10 ± 13.68 ^##^	81.57 ± 16.45 ^##^
MG + succinate	94.36 ± 10.80	87.82 ± 8.56	101.54 ± 8.26	86.37 ± 3.40 *
MG + TPGs	63.49 ± 4.82 **	82.31 ± 9.63	79.94 ± 4.00 **	58.91 ± 6.33 **

Notes: Compared with the NG, ^##^ *p* < 0.01; compared with the MG, ** *p* < 0.01, * *p* < 0.05. NG, normal control group; MG, model control group; TPGs, total polysaccharide and glycoside treatment group.

**Table 3:** Grouping and treatments of intestinal DCs (Each group was stimulated with LPS (1 μg/mL) for 24 h).

Group	Treatment	Concentration
NG + Succinate	Succinate	600 μM
NG + GPR91 siRNA NC	GPR91 siRNA negative control	100 nM
NG + GPR91 siRNA	GPR91 siRNA	100 nM
NG + GPR91 siRNA + Succinate	GPR91 siRNA + Succinate	100 nM + 600 μM

Note: NG, normal control group. GPR91 siRNA NC, negative control siRNA.

## Data Availability

The original contributions presented in this study are included in the article/Supplementary Material. Further inquiries can be directed to the corresponding author.

## Supplementary Materials

The following supporting information can be downloaded at: https://www.mdpi.com/article/10.3390/ph19040606/s1, Table S1. Body weight of mice. Figure S1. Schematic flowchart of the extraction and purification process for TPGs (Total Polysaccharides and Glycosides) from *Aralia echinocaulis*.

## Abbreviations

Rheumatoid arthritis, RA; total polysaccharides and glycosides, TPGs; dendritic cell, DC; tumor necrosis factor-α, TNF-α; interleukin, IL; tricarboxylic acid, TCA; collagen-induced arthritis, CIA; model control group, MG; model + succinate group, MG + succinate; model + TPG treatment group, MG + TPGs; normal control group, NG, normal control + succinate group, NG + succinate; interferon-γ, IFN-γ; myeloperoxidase, MPO; major histocompatibility complex class II, MHC-II); cluster of differentiation 80, CD80; cluster of differentiation 86, CD86; regulatory T, Treg; mature dendritic cells, mDCs; immature DCs, iDCs; forkhead box P3, Foxp3; biologic disease-modifying antirheumatic drugs, bDMARDs; targeted synthetic disease-modifying antirheumatic drugs, tsDMARDs.
