# Supplementary material for: Polysaccharides and Glycosides from Aralia echinocaulis Modulate Succinate Levels in the Gut to Target Intestinal Dendritic Cells via the Receptor GPR91 in the Treatment of Rheumatoid Arthritis
Source: Pharmaceuticals (Basel). 2026 Apr 9;19(4):606. doi: 10.3390/ph19040606 (PMC13119086; doi:10.3390/ph19040606)
Supplement: Supplementary file 1 [file pharmaceuticals-19-00606-s001.zip › pharmaceuticals-4218759-supplementary.pdf]

Supplementary Material

Contents:

Table S1. Body weight of mice

Figure S1. Schematic flowchart of the extraction and purification process for TPG (Total Polysaccharides and Glycosides) from *Aralia echinocaulis*.

Table S1. Body weight of mice (mean ± SD, g, n = 6)

Group	6d	12d	18d	24d
NG	24.82 ± 0.96	25.52 ± 0.72	25.67 ± 0.61	25.87 ± 0.57
MG	23.15 ± 0.67	23.40 ± 1.58	23.78 ± 0.85	24.25 ± 0.81
NG + Succinate	24.31 ± 1.13	24.45 ± 1.32	24.31 ± 1.15	24.95 ± 1.21
MG + TPG	23.42 ± 0.61	23.91 ± 0.66	24.28 ± 0.80	24.70 ± 0.54
MG + Succinate	22.02 ± 0.66	22.98 ± 0.63	22.82 ± 0.63	23.27 ± 1.19

Note: NG, normal group; MG, model group; TPG, total polysaccharides and glycosides

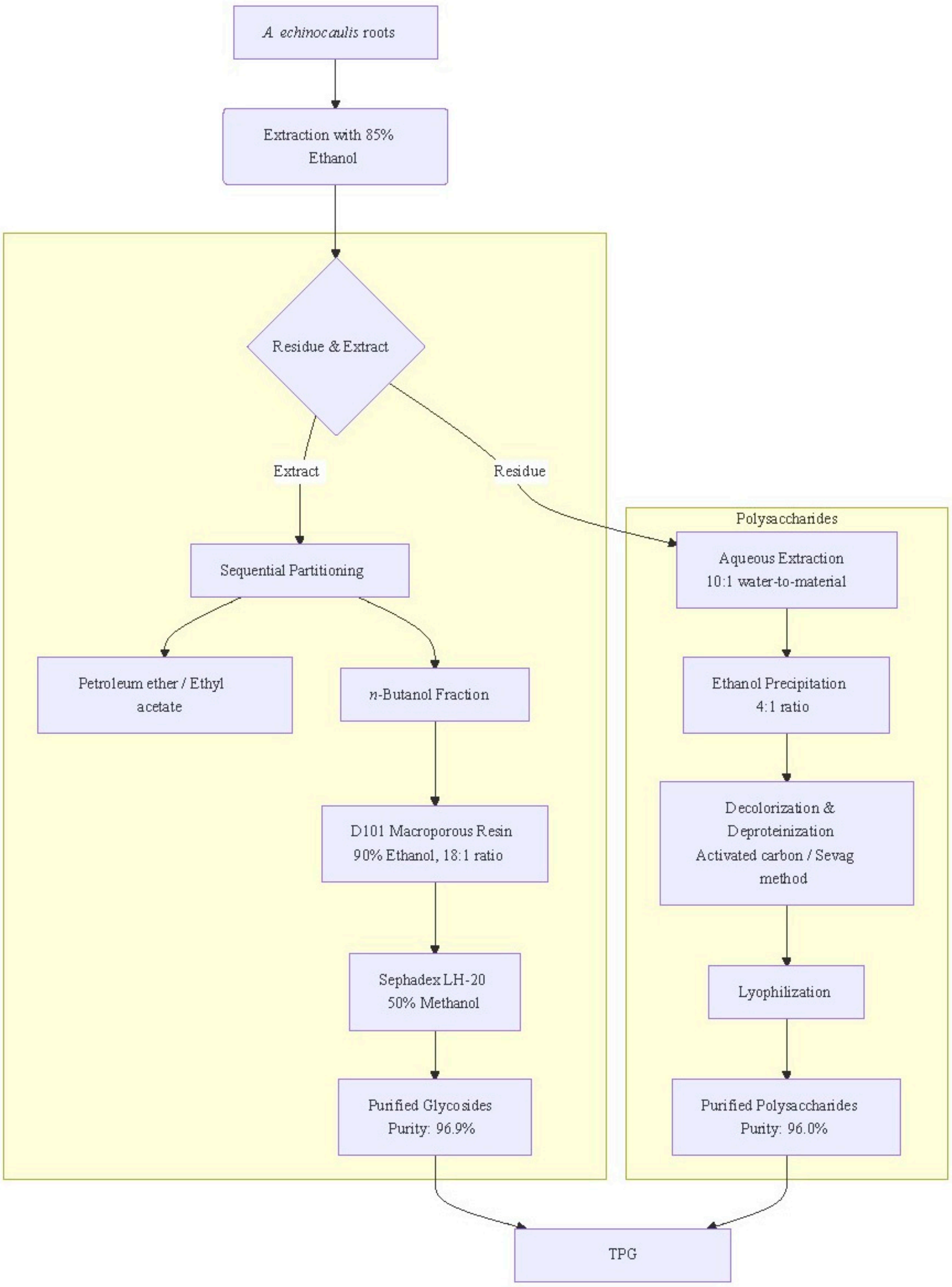

Figure S1. Schematic flowchart of the extraction and purification process for TPG (Total Polysaccharides and Glycosides) from *Aralia echinocaulis*.
